# C-reactive protein cut-off for early tocilizumab and dexamethasone prescription in hospitalized patients with COVID-19

**DOI:** 10.1038/s41598-022-08882-x

**Published:** 2022-03-28

**Authors:** Ana M. Camon, Rodrigo Alonso, Francisco J. Muñoz, Celia Cardozo, Javier Bernal-Maurandi, Laia Albiach, Daiana Agüero, M. Angeles Marcos, Juan Ambrosioni, Marta Bodro, Mariana Chumbita, Lorena De la Mora, Nicole Garcia-Pouton, Gerard Dueñas, Marta Hernandez-Meneses, Alexy Inciarte, Genoveva Cuesta, Fernanda Meira, Laura Morata, Pedro Puerta-Alcalde, Verónica Rico, Sabina Herrera, Montse Tuset, Pedro Castro, Sergio Prieto-González, Alex Almuedo, José Muñoz, Josep Mensa, Gemma Sanjuan, J. M. Nicolas, Ana Del Rio, Jordi Vila, Felipe García, José Antonio Martínez, Carolina Garcia-Vidal, Alex Soriano, Hospital Clinic of Barcelona COVID-19 Research Group, J. L. Blanco, J. Mallolas, E. Martínez, M. Martínez, J. M. Miró, A. Moreno, M. Solá, A. Ugarte, Ana Gonzalez-Cordón, Montse Laguno, Lorna Leal, John Rojas, Berta Torres, S. Fernandez, A. Tellez, F. Fuentes, M. Ayala, E. Sancho, D. Campubri, M. T. de Alba, M. Fernandez, E. Ferrer, B. Grau, H. Marti, M. Muelas, M. J. Pinazo, N. Rodriguez, M. Roldan, C. Subira, I. Vera, N. Williams, A. Almuedo-Riera, A. Aldea, M. Camafort, J. Calvo, A. Capdevila, F. Cardellach, I. Carbonell, E. Coloma, A. Foncillas, R. Estruch, M. Feliu, J. Fernández-Solá, I. Fuertes, C. Gabara, I. Grafia, A. Ladino, R. López-Alfaro, A. López-Soto, I. Macaya, F. Masanés, A. Matas, M. Navarro, J. Marco- Hernández, L. Miguel, J. C. Milisenda, P. Moreno, J. Naval, D. Nicolás, H. Oberoi, J. Padrosa, M. Pellicé, J. Ribot, O. Rodríguez-Núnez, E. Sacanella, F. Seguí, C. Sierra, A. Tomé, M. Torres, H. Ventosa, C. Zamora-Martínez, M. Almela, M. Alvarez, J. Bosch, J. Costa, G. Cuesta, B. Fidalgo, J. Gonzàlez, F. Marco, S. Narvaez, C. Pitart, E. Rubio, A. Vergara, M. E. Valls, Y. Zboromyrska, E. López

**Affiliations:** 1 Department of Infectious Diseases, Hospital Clinic of Barcelona-IDIBAPS, University of Barcelona, C/ Villarroel 170, 08036 Barcelona, Spain; 2 Department of Microbiology, Hospital Clinic of Barcelona, Barcelona, Spain; 3 University of Barcelona, Barcelona, Spain; 4 Institute for Global Health (ISGlobal), Barcelona, Spain; 5 Department of Pharmacy, Hospital Clínic, IDIBAPS, University of Barcelona, Barcelona, Spain; 6 Medical Intensive Care Unit, Hospital Clinic, IDIBAPS, University of Barcelona, Barcelona, Spain; 7 Department of Internal Medicine, Hospital Clinic, IDIBAPS, University of Barcelona, Barcelona, Spain; 8 Department of International Health, Hospital Clinic of Barcelona, Barcelona, Spain; 9 Computer System Unit, Hospital Clinic, Barcelona, Spain; 10 Department of Infectious Diseases, Hospital Clinic of Barcelona, Barcelona, Spain; 11 Medical Intensive Care Unit, Hospital Clinic of Barcelona, Barcelona, Spain; 12 Department of International Health, Hospital Clinic of Barcelona, Barcelona, Spain; 13 Department of Internal Medicine, Hospital Clinic of Barcelona, Barcelona, Spain; 14 Department of Microbiology, Hospital Clinic of Barcelona, Barcelona, Spain; 15 Department of Pharmacy, Hospital Clinic of Barcelona, Barcelona, Spain

**Keywords:** Immunology, Microbiology, Biomarkers, Diseases, Health care, Medical research

## Abstract

Dexamethasone and tocilizumab have been associated with reduction in mortality, however, the beneficial effect is not for all patients and the impact on viral replication is not well defined. We hypostatized that C-reactive protein (CRP) could help in the identification of patients requiring anti-inflammatory therapy. Patients admitted for > 48 h in our hospital for a confirmed or suspected infection by SARS-CoV-2 from February 2020 to February 2021 were retrospectively evaluated. The primary outcome was mortality at 30 days. Demographics and the most relevant variables related with the outcome were included. CRP was stratified by percentiles. Univariate and multivariate analysis were performed. A total of 3218 patients were included with a median (IQR) age of 66 (74–78) years and 58.9% were males. The rate of intensive care unit admission was 24.4% and the 30-day mortality rate was 11.8%. Within the first 5 days from admission, 1018 (31.7%) patients received dexamethasone and 549 tocilizumab (17.1%). The crude analysis showed a mortality reduction in patients receiving dexamethasone when CRP was > 13.75 mg/dL and > 3.5 mg/dL for those receiving tocilizumab. Multivariate analysis identified the interaction of CRP > 13.75 mg/dL with dexamethasone (OR 0.57; CI 95% 0.37–0.89, *P* = 0014) and CRP > 3.5 mg/dL with tocilizumab (0.65; CI95%:0.44–0.95, *P* = 0.029) as independent predictors of mortality. Our results suggest that dexamethasone and tocilizumab are associated with a reduction in mortality when prescribed to patients with a certain inflammatory activity assessed by C-reactive protein.

## Introduction

Severe acute respiratory syndrome coronavirus 2 (SARS-CoV-2) infection has been detected around the world with more than 4 million related deaths^1^. A recent analysis of 44.415 confirmed cases in China described that 81% were asymptomatic or have a mild disease, 14% have a severe disease and 5% a critical disease with an overall mortality of 2.3%^2^. However, among patients that require hospitalization, the mortality rate is around 20%^3^. The activation of the immune system includes the production of type-I interferon (IFN) mainly by plasmacytoid dendritic cells (pDC), however, coronaviruses, and particularly SARS-CoV-2, can suppress type-I INF response by different pathways^4^. Patients with mild to moderate COVID-19 are characterized by adequate type-I INF response while severe cases have low serum levels of type I INF, high viral load, and a dysregulated immune response with persistent hypercytokinemia and dysfunctional T cell response leading to acute distress respiratory syndrome (ARDS)^5^. In this scenario, anti-inflammatory therapies are considered a cornerstone of the management of COVID-19, but its impact on viral replication control particularly during the first days of infection is still a matter of debate.

The most widely evaluated anti-inflammatory drugs are corticosteroids^6,7^ and tocilizumab^8^. It is reasonable to hypothesize that CRP, that have demonstrated a potent capacity to predict the outcome of COVID-19 patients^9^, could be a good parameter to decide when to start an anti-inflammatory therapy. Indeed, some preliminary results showed that IL-6 or CRP serum levels are useful to identify patients that benefit the most from tocilizumab or dexamethasone treatment^10–13^. The correct selection of patients requiring anti-inflammatory therapy is important since not all patients had a beneficial effect^7^, these drugs have adverse effects, and could potentially favour viral replication.

The aim of the present study was to evaluate the outcome of patients receiving dexamethasone or tocilizumab within the first 5 days from hospital admission in a large cohort of COVID-19 patients treated during the first year of pandemic in a tertiary hospital in Barcelona, and to identify potential cut-off points of CRP that predict the response to both anti-inflammatory therapies.

## Methods

### Study design and patients

This observational cohort study was performed at Hospital Clinic in Barcelona (Spain), a 700-bed university centre that provides care for an urban population of 500,000 adults. All patients admitted for ≥ 48 h with confirmed acute SARS-Cov-2 infection by rRT- PCR performed on nasopharyngeal and throat swabs or with a clinical picture highly suggestive of COVID-19 between February 18th, 2020 and February 24th, 2021 were included. Deaths occurring within the first 48 h were included in the analysis. The Institutional Ethics Committee of Hospital Clinic of Barcelona approved the study and due to the nature of the retrospective data review, waived the need for informed consent from individual patients (HCB/2020/0273).

### Data collection

Data were retrospectively collected for all patients included in the study from the electronic health records (EHR). An intelligent system was used to retrieve the high- quality data from EHRs (SILDv1.0 system, S34M^*@*^) as previously described^14^. Variables included were age, sex, co-morbidities (hypertension, chronic heart disease, diabetes mellitus, chronic liver disease, chronic kidney disease, chronic obstructive pulmonary disease, haematological neoplasia, and solid neoplasia), respiratory rate and ambient air arterial Oxygen saturation (SaO_2_) measured with a pulse oximeter at admission, creatinine, lymphocyte count, C-reactive protein (CRP), and lactate dehydrogenase (LDH) within the first 24 h from hospital admission, the need of intensive care (ICU) admission and invasive mechanical ventilation (IMV). Treatment with dexamethasone or tocilizumab within the first 5 days from admission was gathered. This timeframe was selected to mimic the prescription of both drugs in the RECOVERY trials (IQR from 1 to 5 days for both)^7,15^. The dose of dexamethasone was 8 mg/24 h intravenously for 10 days and for tocilizumab a single dose of 600 mg intravenously (400 mg for < 65 kg and 800 mg for > 90 kg). Remdesivir administration was included in the analysis as the only antiviral agent approved for the treatment of COVID-19. The primary outcome was mortality at 30 days.

### Statistical analysis

Categorical variables were described using the absolute number and percentage and continuous variables were dichotomized according to their medians. Categorical variables were compared using a Chi-squared test or Fisher exact test when necessary. For multivariable analysis, variables with a *P* value ≤ 0.05 in the univariable analysis were subjected to further selection by using a backwards logistic regression method. Interactions between dexamethasone or tocilizumab treatment and CRP and other variables were explored. The calibration of the model was assessed by means of the Hosmer–Lemeshow goodness-of-fit test and the area under the receiver operating characteristic (ROC) curve was used to measured predictive ability of the model. Statistical significance was defined as a two-tailed *P* value < 0.05. For interactions included in the final multivariate model, the adjusted OR (95%CI) of the covariate of interest (i.e., dexamethasone or tocilizumab exposure) in each strata of the corresponding risk factor (i.e., CRP group) was calculated according to the formula described by Kleinbaum et al^16^. The analysis was performed by using SPSS version 26 (SPSS Inc., Chicago, IL).

## Results

### Study population

The population evaluated consisted of 3218 patients who were admitted to our hospital during the pandemic. The median (IQR) age was 66 (54–78) years and 58.9% were males. The most common co-morbidities were hypertension (46.3%), chronic heart disease (26.4%), chronic pulmonary disease (24.3%), diabetes mellitus (20.1%), solid neoplasia (15.6%), and chronic renal failure (12.3%). A total of 784 patients were admitted to the ICU (24.4%) and 330 required IMV (10.3%). Regarding therapeutic strategies, 549 (17.1%) patients received remdesivir, and within the first 5 days from admission 1018 (31.7%) received dexamethasone and 549 (17.1%) tocilizumab. The global 30-day mortality rate was 11.8%, and the characteristics associated with mortality are shown in Table 1.

**Table 1:** Characteristics of patients according to the primary outcome (mortality at 30 days).

Variable	Alive (N = 2835)	Dead (N = 381)	*P* value
**Demographics and co-morbidity (%)**			
Age > 66 years	1236 (43.8)	331 (86.9)	< 0.001
Male sex	1642 (58.2)	242 (63.5)	0.049
Chronic heart disease	638 (22.5)	210 (55.1)	< 0.001
Diabetes mellitus	529 (18.7)	117 (30.7)	< 0.001
Haematological disease	165 (5.8)	41 (10.8)	< 0.001
Chronic kidney disease	266 (9.4)	130 (34.1)	< 0.001
Chronic liver disease	203 (7.2)	32 (8.4)	0.383
Hypertension	1228 (43.3)	262 (68.8)	< 0.001
Solid tumour	395 (13.9)	108 (28.3)	< 0.001
Solid organ transplantation	62 (2.2)	13 (3.4)	0.137
HIV infection	35 (1.2)	6 (1.6)	0.623
Chronic lung disease	651 (23)	130 (34.1)	< 0.001
**Clinical characteristics and biomarkers at admission (%)**			
Temperature > 37ºC	1356 (48.6)	130 (28.2)	< 0.001
Oxygen saturation > 94%	1346 (48.4)	98 (28.7)	< 0.001
LDH > 305 U/mL	1283 (48)	210 (62.7)	< 0.001
Creatinine > 0.92 mg/dL	1280 (45.5)	284 (76.5)	< 0.001
Lymphocyte count > 800 cells/mm^3^	1444 (51.3)	134 (36.1)	< 0.001
C-reactive protein > 7.52 mg/dL	1323 (47.5)	248 (68.3)	< 0.001
**Critical support and treatment (%)**			
Intensive Care Unit admission	661 (23.3)	123 (32.3)	< 0.001
Invasive mechanical ventilation	267 (9.4)	63 (16.5)	< 0.001
Remdesivir	519 (18.3)	30 (7.9)	< 0.001
Dexamethasone the first 5 days	882 (31.1)	136 (35.7)	0.071
Tocilizumab the first 5 days	495 (17.5)	54 (14.2)	0.109

HIV: Human Immunodeficiency Virus.

### Outcome

**Figure 1:**
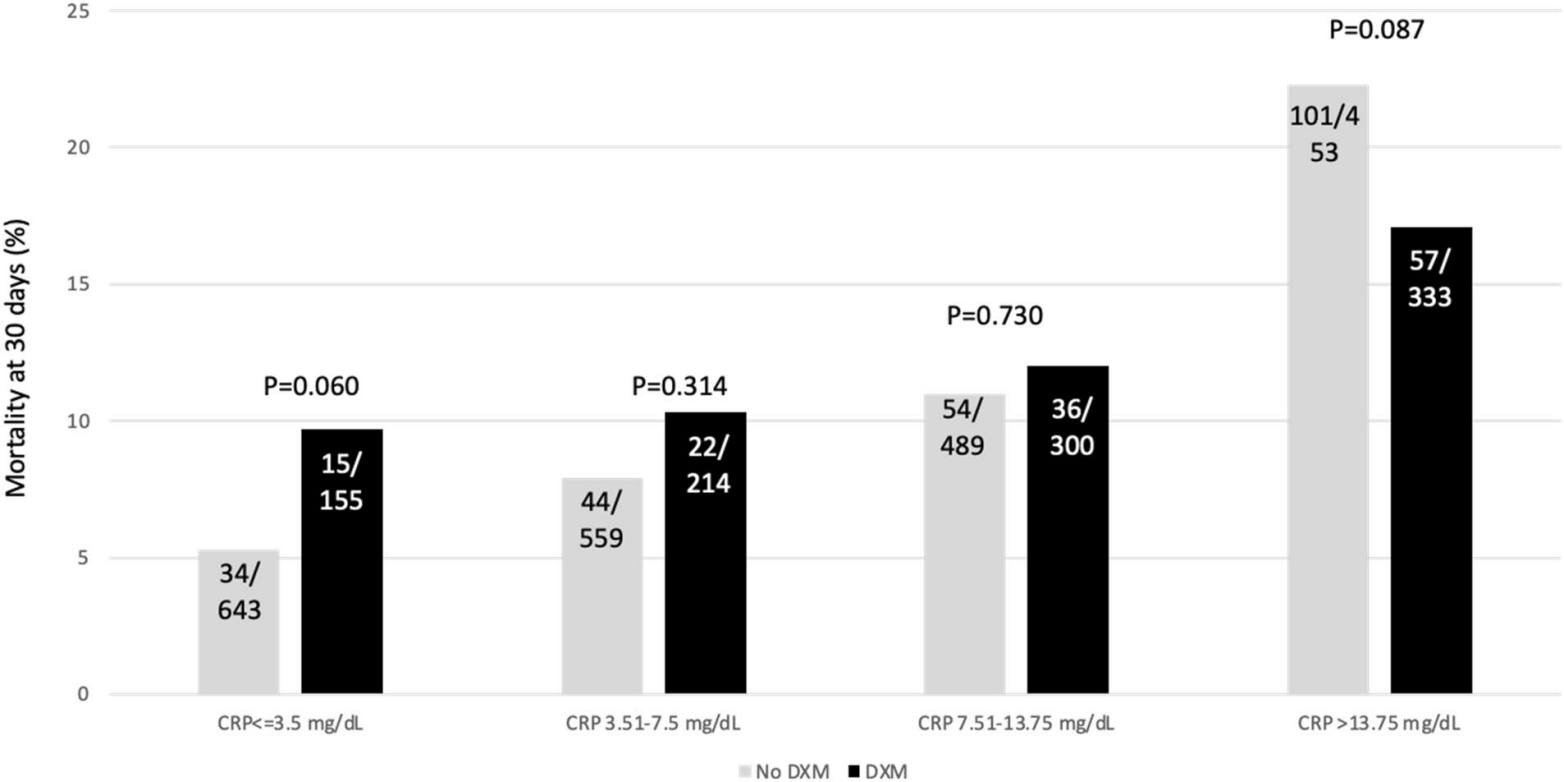
Mortality rate at 30 days in patients receiving or not dexamethasone (DXM) within the first 5 days from admission and stratified by the C-reactive protein (CRP) percentiles (fractions within the bars represents the number of dead patients/total number of patients in this category).

**Figure 2:**
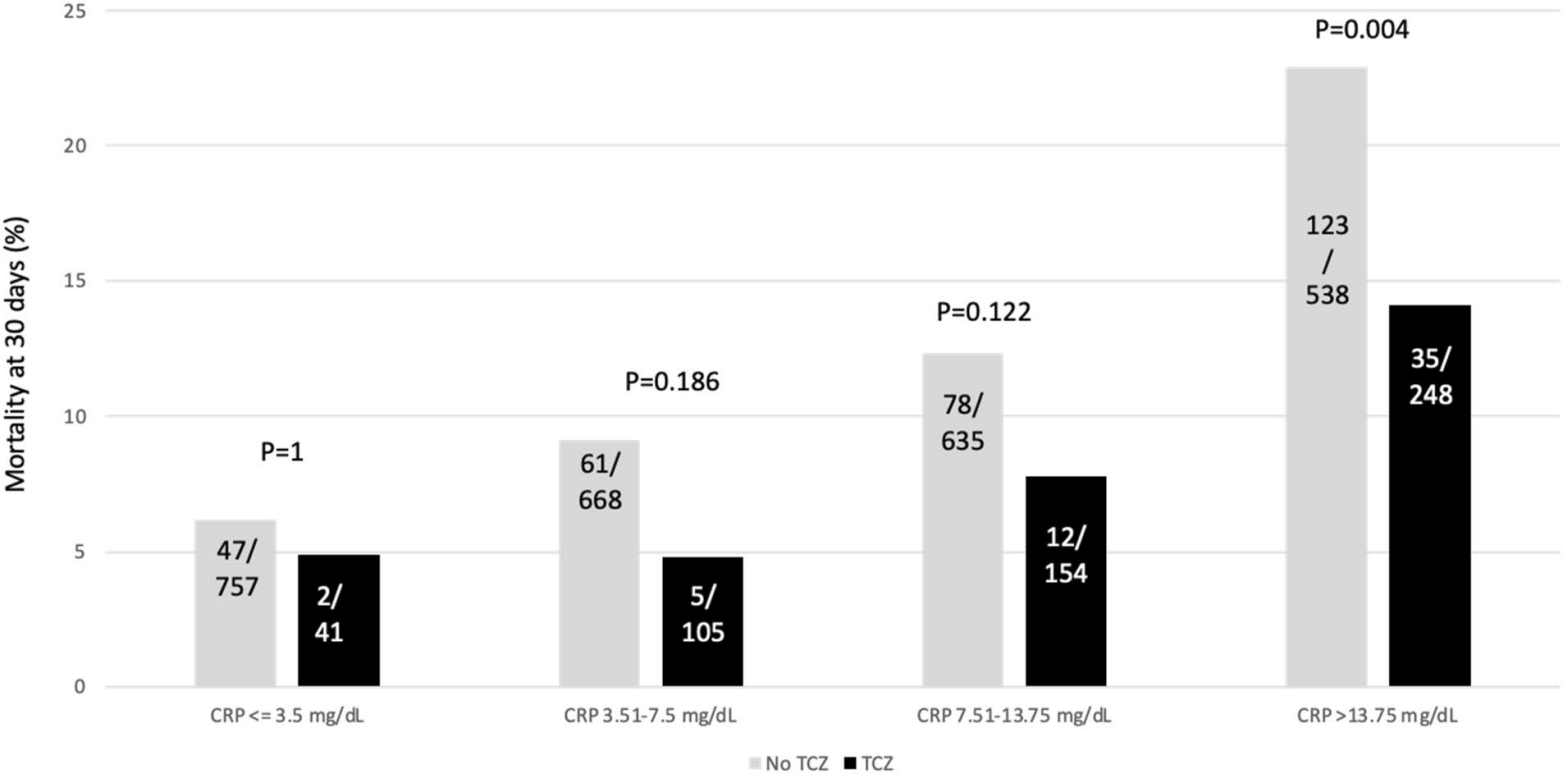
Mortality rate at 30 days in patients receiving or not tocilizumab (TCZ) within the first 5 days from admission and stratified by the C-reactive protein (CRP) percentiles (fractions within the bars represents the number of dead patients/total number of patients in this category).

We explored the mortality among patients that received dexamethasone or tocilizumab according to the CRP percentiles at admission. Figure 1 shows the results for dexamethasone and illustrates that the mortality rate was numerically higher in patients with a CRP ≤ 3.50 mg/dL (percentile 25) if treated with dexamethasone (*P* = 0.06, Chi-square test), similar in those with CRP between 3.51 and 13.75 mg/dL, and numerically lower in patients treated with dexamethasone among those with a CRP > 13.75 mg/dL (*P* = 0.08, Chi-square test). Regarding tocilizumab (Fig. 2), no difference in mortality was observed in patients with a CRP ≤ 3.50 mg/dL; however, in those with a CRP > 3.50 mg/dL there was a numerically lower mortality rate associated with the receipt of tocilizumab, and this difference achieved statistical significance when CRP was > 13.75 mg/dL (*P* = 0.004, Chi-square test).

### Risk factors for mortality

We assessed risk factors associated with 30-days mortality by a multivariate analysis. In this analysis, we included the interaction between dexamethasone or tocilizumab and CRP dichotomized by percentiles. Age > 66 years, co-morbidity, clinical condition (oxygen saturation and fever), creatinine levels, lymphocyte count, invasive mechanical ventilation, treatment with remdesivir, and the interactions between dexamethasone and CRP (cut-off 13.75 mg/dL) and between tocilizumab and CRP (cut-off of 3.5 mg/dL) were retained in the model as independent predictors of mortality (Table 2). The *p* value of the Hosmer–Lemeshow goodness of fit test was > 0.05, and the area under the ROC curve was 0.873 (95% CI 0.851–0.896, *P* = 0.0001) showing a good ability to predict mortality at 30 days. The separate adjusted OR (95% CI) of the association of tocilizumab and dexamethasone with 30-day mortality within each stratum of the corresponding cut-off-defined CRP variables are shown in Table 3. In both cases, over the proposed CRP cut-off points, treatment with either tocilizumab or dexamethasone was significantly associated with a reduction in the mortality rate. Below the proposed cut-offs, the ORs for mortality were not significant.

**Table 2:** Independent variables associated with mortality at 30 days.

Variable	OR (95%CI)	*P* value
Age > 66 years	4.961 (3.367–7.307)	0.001
Chronic heart disease	1.629 (1.213–2.188)	0.001
Haematological disease	1.868 (1.197–2.915)	0.006
Chronic kidney disease	2.392 (1.706–3.354)	0.001
Solid tumour	1.459 (1.067–1.996)	0.018
Temperature > 37 °C	0.716 (0.541–0.947)	0.019
Oxygen saturation > 94%	0.490 (0.360–0.667)	0.001
Creatinine > 0.92 mg/dL	1.569 (1.134–2.172)	0.007
CRP > 3.5 mg/dL (p25)	1.683 (1.118–2.534)	0.013
CRP > 13.75 mg/dL (p75)	2.487 (1.689–3.661)	0.001
Lymphocyte count > 800 cells/mm^3^	0.711 (0.536–0.944)	0.018
Invasive mechanical ventilation	1.668 (1.046–2.659)	0.032
Remdesivir	0.531 (0.336–0.836)	0.006
CRP > 3.5 mg/dL (p25) by tocilizumab the first 5 days	0.682 (0.464–1.002)	0.052
CRP > 13.75 mg/dL (p25) by dexamethasone the first 5 days	0.435 (0.247–0.766)	0.004

Variables included in the model: age, sex, co-morbidity (Chronic heart diseases, Diabetes mellitus, Haematological disease, Chronic kidney disease, hypertension, Solid tumour and Chronic respiratory disease); LDH, creatinine, C-reactive protein, and lymphocyte count at admission [C-reactive protein was introduced by percentiles as well as the interactions between each percentile and tocilizumab or dexamethasone administration within the first 5 days (both variables were also individually included)]; Temperature, and Oxygen saturation at admission; And the need of intensive care admission and invasive mechanical ventilation.

CRP, C-reactive protein. LDH, Lactate Dehydrogenase. P25-75, percentile 25–75.

**Table 3:** Odds ratios for the 2 strata of C-reactive protein (lower or equal or higher than the cut- off) from the significant interactions identified in the final model (calculated according to reference 16).

C-reactive protein (mg/dL)	OR (95%CI) of mortality for patients receiving tocilizumab versus not receiving it	*P* value	C-reactive protein (mg/dL)	OR (95%CI) of mortality for patients receiving dexamethasone versus not receiving it	*P* value
≤ 3.5	1.42 (0.32–6.74)	0.640	** ≤ 13.75**	1.30 (0.92–1.85)	0.13
> 3.5	0.65 (0.44–0.95)	0.029	** > 13.75**	0.57 (0.37–0.89)	0.014

## Discussion

The current cornerstone of COVID-19 treatment is the anti-inflammatory therapy to halt the inflammatory response triggered by SARS-CoV-2. However, we are far from understanding the exact role of persistent viral replication in the maintenance of immune stimulation and even its responsibility in the immune dysregulation leading to severe COVID-19. Therefore, immunosuppressants could be deleterious and the concept of “one size fits for all” probably is not valid for COVID-19 management.

Our results, suggest that dexamethasone significantly reduces the mortality when the patient has an intense systemic inflammatory response measured as a CRP > 13.75 mg/dL, but at the same time, there was a hint of a possible higher mortality when it was administered in patients with low systemic inflammatory response after adjusting for the major risk factors already described in the literature. This is in line with the results obtained by Keller et al.^12^ showing that glucocorticoid treatment of patients with initial CRP ≥ 20 mg/dL was associated with significantly reduced risk of mortality or mechanical ventilation (odds ratio, 0.23; 95% CI, 0.080–0.70), while glucocorticoid treatment of patients with CRP < 10 mg/dL was associated with significantly increased risk of mortality or mechanical ventilation (OR, 2.64; 95% CI, 1.39–5.03). A similar retrospective study also identified a CRP ≥ 10 mg/dL as the cut-off point that predicts the beneficial effect of steroids^17^. Since high viral load^18^, prolonged viral shedding^19^ and the presence of RNAemia^20^ have been associated with worse outcomes in COVID-19, it seems prudent to adequately select the patients and the timing for using corticosteroids. Indeed, previous experience in viral pneumonia (Influenza virus, SARS- CoV and MERS) showed prolonged viral shedding and worse outcome among those patients receiving corticosteroids^21,22^. Data in SARS-CoV-2 is contradictory, while some authors reported longer viral shedding in corticosteroid group^23,24^, others did not^25^.

On the other hand, tocilizumab showed a beneficial effect among patients with a CRP cut-off point > 3.50 mg/dL, a significantly lower cut-off value in comparison to the one for dexamethasone (CRP > 13.75 mg/dL). As an inhibitor of the IL-6, tocilizumab is a selective immunosuppressor blocking exclusively one of the multiple pathways of the inflammatory response. This could explain that using this drug the potential to hamper the viral replication control by the host immune system is limited. In line with this hypothesis, Masia et al.^26^ reported that after adjustment for the baseline viral load, the use of tocilizumab was not associated with a prolonged viral shedding, and it has been suggested that this is because tocilizumab does not reduce the activity of B lymphocytes. Indeed, it was previously documented that tocilizumab did not reduce the efficacy of influenza vaccine in patients with rheumatoid arthritis^27^. Interestingly, the efficacy of tocilizumab in the RECOVERY study^15^, was demonstrated including only patients with a CRP ≥ 7.5 mg/dL. More recently, a post-hoc analysis of the CORIMUNO- TOCI-I trial showed that the likelihood of suffering the primary end point (non-invasive or invasive ventilation requirement, or death) was also lower in the tocilizumab group (18% vs. 57%), when patients with CRP levels > 15 mg/dL were selected (HR 0.18; 95% CI 0.06–0.59)^28,29^.

Our study has several limitations. The major drawback of our study is the retrospective nature. To reduce the potential bias, we have included in the multivariable analysis all the variables potentially implicated in the mortality to adjust for confounding. Secondly, only those treatments already accepted in the majority of the current guidelines or supported by large clinical trials have been evaluated (remdesivir, dexamethasone and tocilizumab), but not other treatments which definitive role is not yet clarified. Thirdly, some patients received both dexamethasone and tocilizumab and the potential effect of combined treatment was not evaluated. Fourthly, days from symptoms onset to admission or to treatment were not available; however, although the timing is important in COVID-19, our results suggest that inflammatory biomarkers by itself are helpful to prescribe dexamethasone or tocilizumab. Finally, viral load or a surrogate marker of the viral load was not available, and this is a critical information since we hypothesise that a potential harmful effect of immunomodulators in patients with low CRP maybe due to the worse control of viral replication as in other respiratory viruses.

Therefore, we propose to start tocilizumab to patients with CRP > 3.5 mg/dL and consider the addition of dexamethasone only to those cases in whom no clinical or biological improvement is observed in the next 48 h. For patients with a CRP > 13 mg/dL we support the initial use of dexamethasone and in line with the recent RECOVERY study, to combine dexamethasone with tocilizumab. In any case, our data also suggest the need of an antiviral agent, and, consequently, we recommend monitoring, in parallel to inflammatory response, the viral load using the cycle threshold of the real time polymerase chain reaction, the RNAemia or any other available marker.

In conclusion, our results suggest that anti-inflammatory therapy with dexamethasone and tocilizumab are associated with a reduction in mortality when prescribed to patients with a certain inflammatory activity as assessed by C-reactive protein, a cheap and widely available biomarker. Personalized treatment following the cut-off points for prescription of tocilizumab (CRP > 3.50 mg/dL) or dexamethasone (CRP > 13.75 mg/dL) within the first 5 days from hospital admission should be considered.
